# Fitting and validation of an agent-based model for COVID-19 case forecasting in workplaces and universities

**DOI:** 10.1371/journal.pone.0283517

**Published:** 2023-03-23

**Authors:** Vignesh Kumaresan, Niranjan Balachandar, Sarah F. Poole, Lance J. Myers, Paul Varghese, Vindell Washington, Yugang Jia, Vivian S. Lee

**Affiliations:** Verily Life Sciences, South San Francisco, California, United States of America; UPSI: Universiti Pendidikan Sultan Idris, MALAYSIA

## Abstract

COVID-19 forecasting models have been critical in guiding decision-making on surveillance testing, social distancing, and vaccination requirements. Beyond influencing public health policies, an accurate COVID-19 forecasting model can impact community spread by enabling employers and university leaders to adapt worksite policies and practices to contain or mitigate outbreaks. While many such models have been developed for COVID-19 forecasting at the national, state, county, or city level, only a few models have been developed for workplaces and universities. Furthermore, COVID-19 forecasting models have rarely been validated against real COVID-19 case data. Here we present the systematic parameter fitting and validation of an agent-based compartment model for the forecasting of daily COVID-19 cases in single-site workplaces and universities with real-world data. Our approaches include manual fitting, where initial model parameters are chosen based on historical data, and automated fitting, where parameters are chosen based on candidate case trajectory simulations that result in best fit to prevalence estimation data. We use a 14-day fitting window and validate our approaches on 7- and 14-day testing windows with real COVID-19 case data from one employer. Our manual and automated fitting approaches accurately predicted COVID-19 case trends and outperformed the baseline model (no parameter fitting) across multiple scenarios, including a rising case trajectory (RMSLE values: 2.627 for baseline, 0.562 for manual fitting, 0.399 for automated fitting) and a decreasing case trajectory (RMSLE values: 1.155 for baseline, 0.537 for manual fitting, 0.778 for automated fitting). Our COVID-19 case forecasting model allows decision-makers at workplaces and universities to proactively respond to case trend forecasts, mitigate outbreaks, and promote safety.

## Introduction

Since early 2020, the COVID-19 pandemic has resulted in millions of illnesses, hospitalizations, and deaths globally [1–3]. The pandemic has led to restrictions on routine activities, including work and education [4–8]. While many of these interventions have been enacted by governments and public health institutions, employers and university leaders have also managed disruptions to in-person activities while minimizing risks to their employees and/or students. The constantly evolving dynamics around the pandemic, including new SARS-CoV-2 variants, changing vaccination and booster rates, different testing approaches, and varying social behaviors all contribute to further uncertainty.

Epidemiological models for COVID-19 that forecast COVID-19 infections, hospitalizations, and deaths have been critical in guiding public health interventions [9–12], including surveillance testing and social distancing. These models include compartmental models [13–21], agent-based models [22–27], and differential equation models [28–30]. Compartmental models involve categorization into and transition between compartments such as “Susceptible”, “Exposed”, “Infected”, and “Recovered” (SEIR). Multi-group compartment models that include dynamics between a site and its surrounding community account for interactions between different populations and can be used to model the impact of various testing strategies [14]. Differential equation models define aggregate COVID-19 trajectories, such as transitions between infection states, using differential equations. Unlike differential equation models that simulate trajectories in aggregate, agent-based models simulate each individual’s trajectory, such as an individual employee’s transition between COVID-19 infection states. Thus compared to differential equation models, agent-based models are more realistic in capturing the complexity and stochasticity of COVID-19 transmission [31]. A further comparison of these methods is shown in Table 1 below.

**Table 1:** Comparison of related COVID-19 modeling methods.

Modeling Technique	Description	Drawbacks
Compartmental [13–21]	SIR and SEIR models with differential equations to simulate movement between disease states.	• Lack of granularity beyond the country level (besides our previous paper [13]) • Unable to automatically account for changes in disease parameters
Agent-Based [22–27]	Simulation-based models that replicate the movement of individuals in a population, given probabilities for various disease parameters.	• Lack of granularity • No integration with testing data (focused on cases)
Differential Equations [28–30]	Mathematical models that attempt to capture various disease parameters through a set of differential equations.	• Lack of granularity • Parameters were estimated roughly and hypothetically

While many compartmental, differential equation, and agent-based models have been developed for the forecasting of COVID-19 cases at the national, state, county, or city level, it has been shown that COVID-19 models have performed poorly when compared with real-world data [32, 33], in part because of the inability to sufficiently capture the dynamics and complexity of COVID-19 transmission or inaccurate assumptions about unknown or uncertain parameters [32]. Fitting parameters on historical testing data can help to overcome these limitations by allowing the model to learn from rapid changes in the population. This can be done manually by adjusting the parameters until we achieve a close fit on the selected historical time period, or automatically by using an algorithm to adjust the parameters daily based on the best fit to the estimated population prevalence at that time.

Furthermore, there are far fewer models for forecasting of COVID-19 in smaller populations such as within workplaces and universities, and these models often lack comprehensive validation against real-world data [34–36]. This includes prior work on a compartmental SEPAYDR (Susceptible-Exposed-Presymptomatic-Asymptomatic-sYmptomatic-Detected-Recovered) model for the COVID-19 simulation in workplaces and universities [14]. There are a number of published models that do address the aforementioned limitations by incorporating manual or automated parameter fitting approaches to fit and validate a model with real-world data, including an agent-based model by Shamil et al., which incorporated manual parameter fitting to predict COVID-19 infections in Ford County, KS and New York City, NY [25], and an agent-based model by Kerr et al., which incorporated Bayesian approximation for automated parameter fitting to predict COVID-19 infections in King County, WA [22]. However, prior work on systematic parameter fitting and comprehensive validation of a COVID-19 simulation model with real-world data at the workplace or university level remains limited.

As shown in Table 1 above, previous COVID-19 modeling efforts fail to capture the granularity required for smaller populations and are inflexible to rapid changes in disease parameters or testing programs. For universities and employers, both of these limitations prevent accurate forecasting of case trends that can be used for strategic changes in their policies. Our proposed solution in this paper overcomes these limitations by implementing model training on local population (university or workplace) testing data and allowing for flexible parameters that can be modified and computed based on updated knowledge about the disease. For the model training, we were able to use daily testing data to fit the disease parameters and provide estimates for projected cases at the local population level, which is more granular than what previous models were able to achieve. For the disease parameters, our model design was able to take in additional disease (ex. variants) and testing (ex. antigen) variables and provide projections based on these factors, while previous models were static and assumed uniform testing regimens.

Here we present the systematic parameter fitting and validation of an agent-based COVID-19 simulation model with real COVID-19 case data. Our agent-based model is extended from a previously published compartment model that includes both community and site-specific compartments [14], with additional modifications for new variants and vaccination status. We present two systematic parameter fitting approaches: a manual fitting approach that sets initial model parameters based on historical data and an automated fitting approach that involves a search for the set of parameters that result in the best fit based on lookahead prevalence estimation. We validate our approaches with real COVID-19 case data and compare against a baseline scenario where no fitting is used. Our aim is to show that our model can accurately forecast COVID-19 cases across different phases of a pandemic, and to compare a manual and automated approach for fitting and validating the model with real COVID-19 case data from an employer.

## Materials and methods

Our model is an extension of a previously published compartmental SEPAYDR (Susceptible-Exposed-Presymptomatic-Asymptomatic-sYmptomatic-Detected-Recovered) model [14]. We updated the model to account for additional complexity in disease dynamics as described below. Furthermore, our original model did not enable fitting of parameters to real-world data; here we update the model to enable fitting of parameters and evaluation with real-world COVID-19 data from workplaces and universities.

### Data

We utilized testing data from an organization site to build and validate our modeling approaches. During the selected periods, PCR testing was performed once a week for any participants that came on-site and testing data was automatically recorded and collected in a software tool. This tool connected to the testing data from the laboratories and displayed the aggregate numbers back to the organization in a web console, while providing us with the raw laboratory results. In addition to obtaining data on positive tests and tests completed over time, we were able to calculate the number of participants who were eligible for testing on a given day. This combination of data was used to estimate population prevalence for the site. Since all data used for our modeling was aggregated, we did not seek approval from an IRB.

### Model overview

We published the details of our original compartmental model in PLOS ONE [14]. This model was useful for prospective modeling, however in order to integrate the time-varying aspect of certain disease parameters, we needed to add additional complexity to our model. Additionally, a more flexible model allowed us to calculate outcome metrics that were aligned with how our clients were thinking about their risk.

For these reasons, we converted our original compartmental model into an agent-based model that allowed us to simulate individual participants and tests. We achieved this by converting the differential equations in the previous model into probabilities that each agent moves between each compartment in a given day. An additional benefit of the agent-based model is its ability to return multiple simulations and return a range of plausible options for the trajectory of the disease given the model parameters, in contrast to the single forecast provided by the compartmental model. These design improvements were made due to the variability that was unaccounted for in our original compartmental modeling. Specifically, the agent-based design is able to account for factors like disease details (ex. incubation period) and testing parameters (ex. cohorts of interest). The new model design also allowed us to calculate metrics that were more robust, since we were able to observe the output from multiple case trajectories and better capture the uncertainty in our projections. For details on the assumptions and parameters of the model, please refer to the original compartmental model paper [14].

Given the changes in the pandemic since the original model, we also decided to integrate additional variables to take certain factors into account, including vaccination rates (both in the workplace and in the community), reduced vaccine efficacy against variants, and the option for lower-sensitivity antigen testing instead of PCR testing. For details on additional parameters that were included in the model, please refer to S1 File.

### Case trajectory projection

To help clients develop proactive COVID strategies, we projected the future case trajectory for their workplace locations, given what we know about their population and their prevalence. We tested two methods of achieving this: manual parameter fitting and automated parameter fitting. The initial parameters for the simulations were manually set for both approaches through a combination of publicly available community data and client information. For more details on the initial simulation parameters, please refer to S2 File.

Our manual parameter fitting approach involved calculating the client’s empirical weekly test positivity over previous weeks, and then fitting the output of our agent-based model to closely align with that week-over-week positivity. This was largely a qualitative effort that involved adjusting the basic virus reproduction number R0_W_ until we observed a weekly test positivity curve that was similar to what was observed in the historical testing data. Once this was achieved, we were able to make projections for the employer/university’s population for the next 1–2 weeks by observing the weekly test positivity for the remaining days in the simulation.

We built upon this method by implementing automated parameter fitting, which involved calculating the estimated prevalence in the population for each historical day, identifying the simulation candidates that best fit that prevalence trajectory, and using the parameters from that run to initialize the model run for the next day, rather than by directly fitting the parameters. Let $y_{\Phi_{i}}(j)$ denote the modeled trajectory output for simulation candidate *i* on the *j*^*th*^ day of the simulation, where *Φ*_*i*_ is the set of model parameters associated with simulation candidate *i*. Let ${\hat{y}}_{Pr}(j)$ denote the prevalence estimation associated with the ground truth workplace test positivity data for the *j*^th^ day of the simulation. Initially, each candidate simulation candidate *i* is assigned a weight *Ψ*_*i*_(0) of 1/*C* where *C* is the total number of candidate simulations. For each subsequent day *j* of the 14-day fitting period, each candidate simulation candidate *i* is assigned a weight *Ψ*_*i*_(*j*) as follows. The fraction of the workplace population that should test positive on day *j* of simulation candidate *i* is denoted by *F*_*w*_*pos*,*i*_(*j*) and calculated as

$$F_{w\_pos,i}(j)=\frac{I_{w,i}{(j)+D}_{w,i}(j)}{N_{w,i}(j)}$$

where *I*_*w*,*i*_(*j*) refers to the number of predicted infectious individuals in the workplace on day *j*, *D*_*w*,*i*_(*j*) refers to the number of predicted detected (tested positive) individuals in the workplace on day *j*, and *N*_*w*,*i*_(*j*) refers to the total number of individuals in the workplace on day *j*. Then the likelihood *L*_*i*_(*j*) of simulation candidate *i* is calculated using the probability mass function at ${\hat{y}}_{Pr}(j)$, the ground truth number of individuals who tested positive on day *j*, of the binomial distribution $Binom(n={\hat{N}}_{Pr}(j),p=F_{w\_pos,i}(j))$ where ${\hat{N}}_{Pr}(j)$ is the ground truth number of tested individuals on day *j* based on the prevalence estimation:

$$L_{i}(j)=\frac{{\hat{N}}_{Pr}(j)}{{\hat{y}}_{Pr}(j)}F_{w\_pos,i}(j{)}^{{\hat{y}}_{Pr}(j)}\cdot {\left(1-F_{w\_pos,i}(j)\right)}^{\left({\hat{N}}_{Pr}(j)-{\hat{y}}_{Pr}(j)\right)}$$

This ensures that simulation candidates that produce projections closer to the ground truth data will have higher likelihoods. Then the weight for simulation candidate *i* for day *j* is assigned as

$$\Psi_{i}(j)=L_{i}(j)\Psi_{i}(j-1)$$

$$\Psi_{i}(j)=\frac{\Psi_{i}(j)}{\sum_{k=1}^{C}\Psi_{k}(j)}$$

For each subsequent day *j*+1 of the simulation, simulation candidates are sampled based on these weights and the simulation is continued for the sampled candidates. This ensures that candidates with lower likelihood are filtered out over the course of the simulation and ultimately we get candidates with high likelihood at the end of the 14-day automated fitting period.

Once this has been done for all historical data, we can project forward by running the simulation with the latest fitted parameters and observing the trajectory for the next 1–2 weeks. In contrast to the manual approach, this process is fully automated and requires no adjustment.

To achieve this, we needed to first estimate the prevalence in the population based on our knowledge about their testing strategy. We were able to obtain test rationale and risk category for each participant, which we used to create cohorts with equal estimated COVID risk and, more importantly, equal chance of being tested. We used this information to then calculate the estimated prevalence based on the distribution of positive tests among these cohorts. These estimates ultimately served as the ground truth for our automated fitting approach. Working with these prevalence estimates rather than with raw test positivity allowed us to include possibly infectious but untested individuals in the population.

### Outputs

Our agent-based model allows us to capture a variety of outcome metrics, but for the purposes of this paper, we will focus on those related to test positivity. We also found that employers and universities were most interested in using test positivity projections for their decision-making, so we focused our validation on related metrics. To account for daily fluctuations in testing, we centered our modeling around 7 day average test positivity.

To validate our modeling results, we calculated metrics based on the fit of our model projections to the true observed outcome data for that time period. In keeping with previous published work, we will show performance of our manual and automated fitting approaches using Root Mean Squared Log Error (RMSLE). The log calculation in the RMSLE metric penalizes underestimates more severely, which we felt aligned closely with how organizations used these projections (proactive guidance to prevent potential outbreaks). Other comparison metrics were also calculated and can be seen in S1 File.

We also included a baseline model to replicate the previous model state where no fitting was available. In this scenario, we initialized the model based on the community Rt and test positivity and then let it run for the chosen time period without any adjustments. Without any fitting, the model is not able to update projections based on testing data and instead relies on the programmed disease parameters to calculate positivity estimates.

The ultimate goal of this modeling was to enable our clients to make proactive decisions about testing and workplace/university policies. The fit metrics above are important to ensure that we are making accurate projections, but we also made qualitative observations about the test positivity trajectories in comparison to the observed test positivity graphs.

To show the use of our model to aid clients in various situations, we chose to focus on periods of time that covered two different COVID scenarios: an increase in cases and a decrease in cases. For both scenarios, we compared the manual and automated parameter fitting results for both a 7 day lookahead projection and a 14 day lookahead projection. For simplicity, we chose to keep the fitting window at 14 days for all scenarios, but this could be modified in later experiments.

## Results

For each scenario, we compared the 7 day average test positivity from the baseline, simulated, and observed trajectories. Note that the first 7 days of fitting are not shown in the visualizations since a 7 day average cannot be calculated for that time period.

### Scenario 1: Rising prevalence

**Fig 1:**
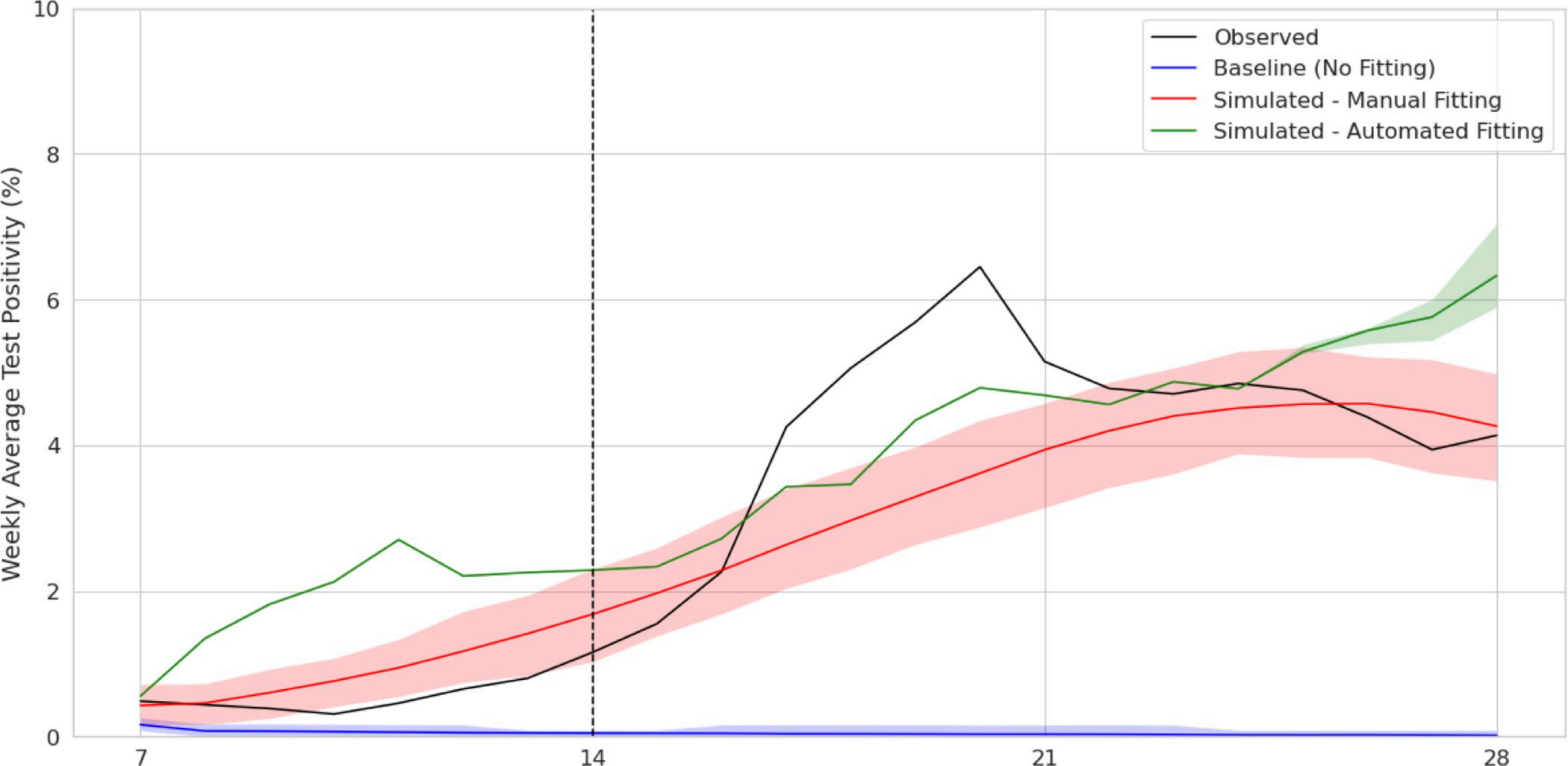
Comparison of weekly average test positivity and percentage change in weekly average test positivity, Scenario 1. Weekly average test positivity and 90% confidence intervals across 100 repeated runs each for baseline (no fitting), manual, and automated parameter fitting, compared with the observed ground truth.

Fig 1 and Table 2 show that both fitting approaches were able to successfully project the increase in cases in the population. The manual fitting accurately projected a rise and subsequent decline in cases, but did not reach the same peak test positivity as was observed in the testing data for the projected period. The automated fitting had more variability but reached the same peak test positivity as the observed data.

**Table 2:** RMSLE for the forecast periods of 7 and 14 days.

Lookahead Period	Baseline (No Fitting)	Automated	Manual
7 days	.02873	.00415	.00664
14 days	.02893	.00399	.00480

Root Mean Log Square Error (RMSLE) for weekly average test positivity predictions compared to observed data in 7 and 14 day lookahead periods in Scenario 1.

Both approaches performed significantly better than the baseline model approach, which in this case was unable to predict any rise in cases. This is expected since the baseline model was set to a relatively low prevalence and Rt at the start of the time period and never received any new data to change from that positivity. In this scenario, the automated fitting model produced consistent projections (tight confidence intervals) until the final few days of the simulation due to candidate filtering resulting in sampling of nearly identical simulation candidates for the first several days of the simulation.

### Scenario 2: Decreasing prevalence

**Fig 2:**
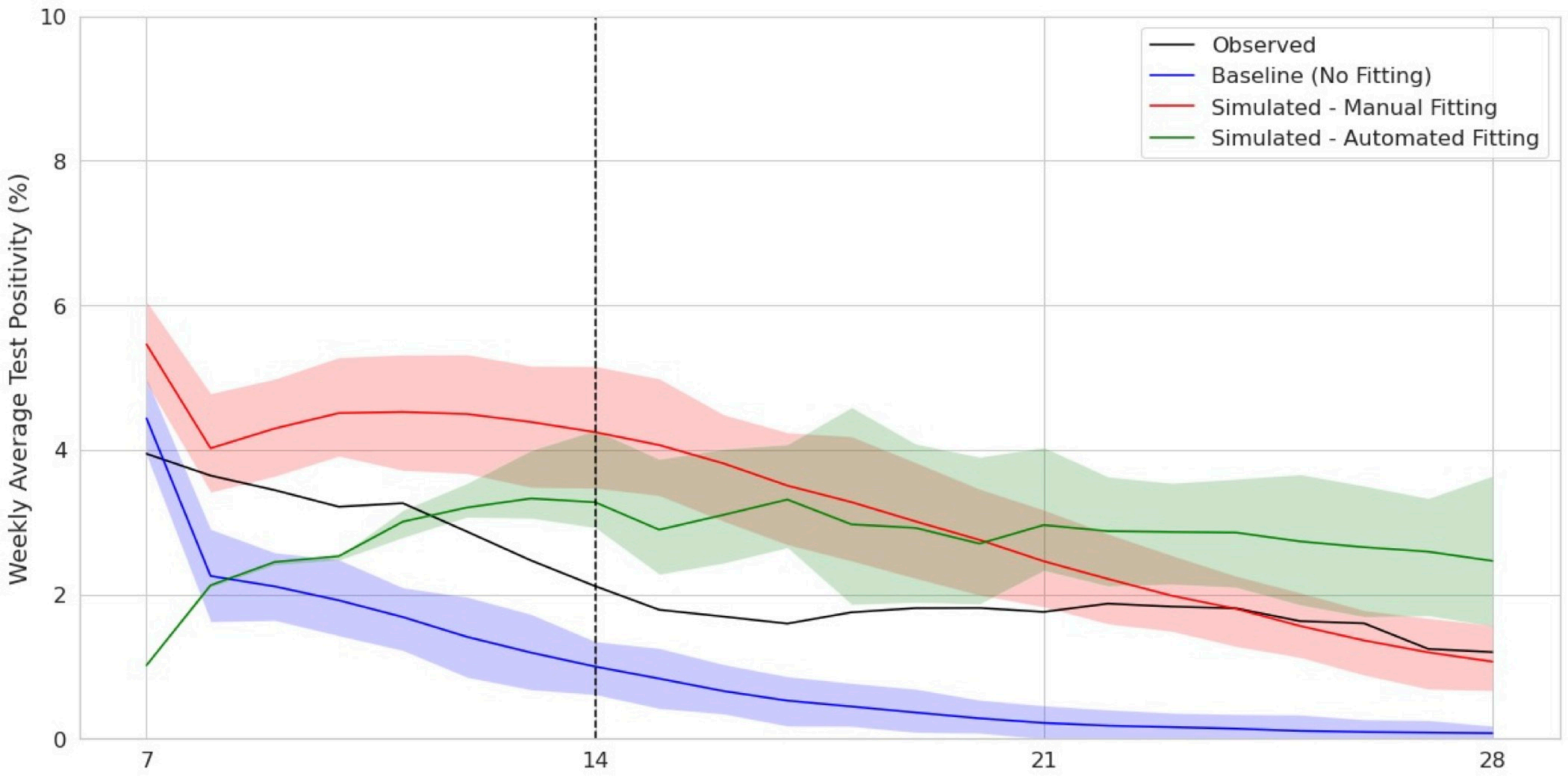
Comparison of weekly average test positivity and percentage change in weekly average test positivity, Scenario 2. Weekly average test positivity and 90% confidence intervals across 100 repeated runs each (panel a) for baseline (no fitting), manual, and automated parameter fitting, compared with the observed ground truth.

Fig 2 and Table 3 show that both fitting approaches successfully projected the decrease in cases. The manual approach predicted a uniform decrease, while the automated approach had an initial rise in positivity during the fitting period that was followed by a subsequent decrease in cases that followed a similar pattern to the observed decrease. D to this initial increase, the automated approach performed worse than the manual approach for both lookahead periods. However, looking at the patterns of the projection trajectories, the automated approach more closely follows the trend in the test positivity, while the manual approach projected a sharper decline in cases.

**Table 3:** RMSLE for the forecast periods of 7 and 14 days.

Lookahead Period	Baseline (No Fitting)	Automated	Manual
7 days	.00924	.00914	.00412
14 days	.01152	.00923	.00750

Root Mean Log Square Error (RMSLE) for weekly average test positivity predictions compared to observed data in 7 and 14 day lookahead periods.

## Discussion

During the COVID-19 pandemic, workplaces and universities are constantly monitoring their population test positivity to make decisions about testing and site policies. We presented the fitting and validation of an agent-based model for projection of test positivity up to 14 days ahead, which we have used as a framework for assisting organizations with these decisions.

While agent-based models have been used in other studies to predict COVID prevalence, our approach includes two key differences: a) our modeling approach includes the community-workplace interaction that was presented in our original compartmental model paper, and b) we were able to validate our manual and automated parameter approaches directly on an organization’s testing data. Previous COVID-19 modeling work has primarily focused on publicly-available testing data at the county or state level in the United States, and prior work on modeling and validation at the workplace or university level has been limited. Vecherin at al. developed a stochastic agent-based microexposure model for COVID-19 risk modeling at workplaces, and demonstrated the efficacy of the model on reproducing an outbreak in a South Korean call center [37]. However, Vecherin at al. did not use separate windows of time for fitting and for validating the model, potentially leading to model overfitting, and did not report metrics for model performance on that real-world scenario [37]. Our work addresses a gap in COVID-19 forecasting models by fitting and validating real-world university or workplace testing data. Our work uses disjoint windows of time for fitting and validation and we report numerical performance metrics for each of our methods.

We compared the manual and automated fitting approaches for the agent-based model through a qualitative comparison of the projection trajectories and a quantitative comparison of error metrics across three different scenarios. Overall, we observed that both approaches are able to accurately project trajectories for both 7-day and 14-day lookahead periods, with the manual fitting approach leading to more uniform trajectories and the automated approach leading to trajectories with more rapid changes. While the manual fitting approach performed better in the decreasing cases scenario (Scenario 2), it failed to capture the magnitude of the spike in the increasing cases scenario (Scenario 1), which is the primary scenario that organizations are concerned about when making strategic decisions. This may be due to the fact that the prevalence estimation algorithm (which is used in the automated fitting process) by design tends to be more conservative and overestimate infectiousness, which would lead to more conservative projections from the automated fitting of the agent-based model.

A benefit of the agent-based model approach compared to the compartmental model used previously is that we have the ability to adjust parameters to show projections based on strategies that an employer or university is considering. Our most common use case of this is to adjust testing parameters such as testing frequency (moving from testing twice a week to testing once a week) or testing sensitivity (moving from PCR to antigen tests), and then view the changes in resulting prevalence trajectories. This scenario planning requires the fitting and validation work that was presented in this paper, and can serve as a useful aid in decision-making.

Our modeling approach has some limitations, primarily driven by assumptions that are necessary but sometimes unclear based on our understanding of the disease and the population of interest. We relied on estimates from the literature for parameters related to disease transmission and testing sensitivity, which need to be continually updated as more is known in the scientific community. Our automated fitting approach relies on continuous prevalence estimates, which themselves require information on tests and test rationale that may not always be available.

We set our projection period to 14 days because of the many factors that could change during the projection period, including movement of individuals into/out of the population, changes in testing or site policies, or the identification of a new variant, which could lead to a divergence in predictions. If one of these events were to occur, the model assumptions would no longer be valid, and the model would therefore be likely to produce inaccurate predictions. To account for any of these events, the initial model parameters would have to be adjusted to reflect the updated knowledge; in the case of a new variant, we would expect to see an increase in test positivity, which, according to our suggested policies, would spur the employer or university to call for an updated fitting and projection. Our model produces accurate 7- and 14-day projections, so the model can be updated weekly or biweekly based on new data to produce accurate predictions on an ongoing basis.

Test positivity is used as the primary metric of interest in our modeling, since we found this to be the main outcome that universities and employers are interested in monitoring and projecting. However, this means that regular testing and monitoring is needed in order for the agent-based model to provide meaningful projections, since shifts in testing rate and the population being tested could lead to differences in observed (and therefore, predicted) test positivity, even if there is no change in the underlying infection dynamics.

In future work, we’re hoping to improve our model’s ability to capture complexity at a workplace or university. Possible modifications include accounting for population changes, multiple circulating variants, and sub-locations/cohorts within a larger site. We would also like to explore the potential value of this type of modeling for other employee health interventions, such as other infectious diseases or potentially chronic disease management. From a technical perspective, we would like to improve our automated fitting procedure by integrating the ability to ‘learn’ additional parameters, such as the workplace R0.

## Conclusion

We presented the fitting and validation of an agent-based COVID-19 forecasting model that has been used to guide COVID-19 decision-making at workplaces and universities. We demonstrated a manual fitting and an automated fitting approach that both outperformed the baseline model with no fitting. Based on the predictions of our models, workplaces and universities can assess the trends in test positivity for the upcoming 7 or 14 days and proactively adjust site safety policies to mitigate positive cases and maximize safety.

We also demonstrated a framework for how to combine quantitative and qualitative metrics to validate our model for a particular population. We found that while the manual approach outperformed the automated approach for the decreasing and stable scenarios, it was unable to capture any rapid fluctuations, which are a priority for organizations looking to make site policy decisions. Thus, we proposed the use of the automated approach due to its ability to capture previous changes in prevalence, and use that information for future projections. Finally, we discussed the need for continued testing data and up-to-date knowledge of worksite policies to ensure accurate modeling, since even small changes in behavior can affect disease transmission dynamics. By integrating this simulation modeling framework into an existing testing program, employers and universities can benefit from early warning signs and take measures to properly maintain the safety of their population. Future work will focus on model improvement through integration of additional disease parameters (like workplace R0) and exploration of the utility of this modeling for other employee health interventions.

## Supporting information

S1 File Modifications to model parameters from original compartmental model. (DOCX)

S2 File Initial parameters used for fitting approaches. (DOCX)

S3 File Full performance metrics. (DOCX)
