# Supplementary material for: Fitting and validation of an agent-based model for COVID-19 case forecasting in workplaces and universities
Source: PLoS One. 2023 Mar 23;18(3):e0283517. doi: 10.1371/journal.pone.0283517 (PMC10035834; doi:10.1371/journal.pone.0283517)
Supplement: S1 File — (DOCX) [file pone.0283517.s001.docx]

# **Supporting information S1: Modifications to model parameters from original compartmental model**

**Vaccination Rate**

With the increased availability of vaccinations, we decided to include vaccination rate as a modifiable parameter for our model. Similar to how we set initial prevalence separately for the workplace and for the community, we also separated out workplace vaccination rate and community vaccination rate, since these may sometimes be dramatically different. For simplicity, we chose to treat vaccinated individuals with the same immunity as individuals who had previously been infected and had recovered from COVID. Community vaccination rates were obtained through public county data from the CDC, while workplace vaccination rates were obtained through conversations with organizations.

**Vaccine Efficacy**

As variants started to become widespread, more information became known about reduced vaccine efficacy. To account for this, we included an additional parameter that allowed for reduction in immunity for all vaccinated individuals in the population.

**Antigen Testing**

Frequent antigen testing became a cost-effective alternative to PCR testing that many organizations considered during the program. To account for this, we created a flag that would indicate if the simulation used antigen testing or not (in which case it would default to PCR testing). This flag determined all tests in the simulation, so we were not able to run experiments with combined antigen and PCR testing. The primary modification was the difference in sensitivity and specificity between the two tests, which were taken from estimates in the literature. Since our model also returned individual test counts, we were able to run cost optimization experiments to provide organizations with information on the tradeoff between PCR and antigen testing for their population.
