# Supplementary material for: Fitting and validation of an agent-based model for COVID-19 case forecasting in workplaces and universities
Source: PLoS One. 2023 Mar 23;18(3):e0283517. doi: 10.1371/journal.pone.0283517 (PMC10035834; doi:10.1371/journal.pone.0283517)
Supplement: S2 File — (DOCX) [file pone.0283517.s002.docx]

# **Supporting information S2: Initial parameters used for fitting approaches**

| **Parameter** | **Value** |
| --- | --- |
| Length of modeling simulation (days) | 28 (14 for fitting, 14 for projection) |
| Testing Type and Frequency | 1 PCR test per employee per week |
| Initial community case prevalence | Taken from community county data |
| Initial workplace case prevalence | Taken from prevalence estimation algorithm |
| Initial community vaccination rate | Taken from community county data |
| Initial workplace vaccination rate | Taken directly from organization |
