# Supplementary material for: Fitting and validation of an agent-based model for COVID-19 case forecasting in workplaces and universities
Source: PLoS One. 2023 Mar 23;18(3):e0283517. doi: 10.1371/journal.pone.0283517 (PMC10035834; doi:10.1371/journal.pone.0283517)
Supplement: S3 File — (DOCX) [file pone.0283517.s003.docx]

# **Supporting information S3: Full performance metrics**

Metric definitions:

MSE: Mean Squared Error

RMSE: Root Mean Squared Error

MSLE: Mean Squared Log Error

RMSLE: Root Mean Squared Log Error

MAE: Mean Absolute Error

CoV: Coefficient of Variation

**Scenario 1: Rising Prevalence**

| **Lookahead Period** | **Baseline (No Fitting)** | **Automated** | **Manual** |
| --- | --- | --- | --- |
| 7 days | MSE: .17304  RMSE: .04160  MSLE: .08256  RMSLE: .02873  MAE: .03899  CoV: .00925 | MSE: .01619  RMSE: .01272  MSLE: .00172  RMSLE: .00415  MAE: .00961  CoV: .00283 | MSE: .03565  RMSE: .01888  MSLE: .00441  RMSLE: .00664  MAE: .01657  CoV: .00420 |
| 14 days | MSE: .16345  RMSE: .04043  MSLE: .08368  RMSLE: .02893  MAE: .03904  CoV: .00898 | MSE: .01734  RMSE: .01317  MSLE: .00159  RMSLE: .00399  MAE: .01016  CoV: .00292 | MSE: .01881  RMSE: .01372  MSLE: .00231  RMSLE: .00480  MAE: .01021  CoV: .00305 |

**Scenario 2: Decreasing Prevalence**

| **Outcome** | **Baseline (No Fitting)** | **Automated** | **Manual** |
| --- | --- | --- | --- |
| 7 days | MSE: .00699  RMSE: .00836  MSLE: .00855  RMSLE: .00924  MAE: .00824  CoV: .00467 | MSE: .03067  RMSE: .01751  MSLE: .00835  RMSLE: .00914  MAE: .01557  CoV: .00977 | MSE: .00368  RMSE: .00607  MSLE: .00170  RMSLE: .00412  MAE: .00523  CoV: .00339 |
| 14 days | MSE: .00851  RMSE: .00922  MSLE: .01243  RMSLE: .01115  MAE: .00904  CoV: .00532 | MSE: .02241  RMSE: .01497  MSLE: .00852  RMSLE: .00923  MAE: .01453  CoV: .00897 | MSE: .01878  RMSE: .01370  MSLE: .00562  RMSLE: .00750  MAE: .01053  CoV: .00790 |

**Explanation of metrics**

We report MSE, RMSE, MSLE, RMSLE, MAE, and CV of rolling averages of predicted test positivity when evaluated against rolling averages of real test positivity. MAE is less sensitive to large errors than RMSE, and RMSLE includes a log calculation that results in larger penalties for underestimates than does RMSE. RMSE and RMSLE are reported with the same unit as the outcome (test positivity or %change in test positivity), whereas MSE and MSLE are reported as the square of these units. Lastly, CV can be useful to evaluate the correlation between the predictions and ground-truth even if there is a discrepancy in the overall magnitude of values. We report each of these evaluation metrics, and we focus on RMSE and RMSLE, because organizations are primarily concerned with predicting and pre-empting potential upcoming outbreaks/rises in cases and with predicting overall trends in cases (e.g. rises/falls).
